# Supplementary material for: Effectiveness of Routine Measurement of Health-Related Quality of Life (HRQOL) in Improving Patient-reported Outcomes in Primary Care Patients with Chronic Knee and Back Problems – A Cluster Randomised Controlled Trial
Source: PLOS Digit Health. 2026 Apr 15;5(4):e0001337. doi: 10.1371/journal.pdig.0001337 (PMC13082660; doi:10.1371/journal.pdig.0001337)
Supplement: S1 Appendix — (DOCX) [file pdig.0001337.s001.docx]

# **S1 Appendix. CONSORT 2010 checklist for randomized trial**

| Section/Topic | Item No | Checklist item | Reported on page No | | |  |
| --- | --- | --- | --- | --- | --- | --- |
| Title and abstract | | | | |  |  |
|  | 1a | Identification as a randomised trial in the title | 1 | | |  |
|  | 1b | Structured summary of trial design, methods, results, and conclusions (for specific guidance see CONSORT for abstracts) | 2 | | |  |
| Introduction | | | | |  |  |
| Background and objectives | 2a | Scientific background and explanation of rationale | 4, 5 | | |  |
|  | 2b | Specific objectives or hypotheses | 4, 5 | | |  |
| Methods. | | | | |  |  |
| Trial design | 3a | Description of trial design (such as parallel, factorial) including allocation ratio | 5 | | |  |
|  | 3b | Important changes to methods after trial commencement (such as eligibility criteria), with reasons | Nil | | |  |
| Participants | 4a | Eligibility criteria for participants | 7 | | |  |
|  | 4b | Settings and locations where the data were collected | 6, 7 | | |  |
| Interventions | 5 | The interventions for each group with sufficient details to allow replication, including how and when they were actually administered | 6, 7 | | |  |
| Outcomes | 6a | Completely defined pre-specified primary and secondary outcome measures, including how and when they were assessed | 5 | | |  |
|  | 6b | Any changes to trial outcomes after the trial commenced, with reasons | Nil | | |  |
| Sample size | 7a | How sample size was determined | 6 | | |  |
|  | 7b | When applicable, explanation of any interim analyses and stopping guidelines | 8 (Ref 26) | | |  |
| Randomisation: |  |  |  | | |  |
| Sequence generation | 8a | Method used to generate the random allocation sequence | 6 | | |  |
|  | 8b | Type of randomisation; details of any restriction (such as blocking and block size) | 6 (Ref 26) | | |  |
| Allocation concealment mechanism | 9 | Mechanism used to implement the random allocation sequence (such as sequentially numbered containers), describing any steps taken to conceal the sequence until interventions were assigned | 6 (Ref 26) | | |  |
| Implementation | 10 | Who generated the random allocation sequence, who enrolled participants, and who assigned participants to interventions | 6 | | |  |
| Blinding | 11a | If done, who was blinded after assignment to interventions (for example, participants, care providers, those assessing outcomes) and how | 6 | | |  |
|  | 11b | If relevant, description of the similarity of interventions | 6 | | |  |
| Statistical methods | 12a | Statistical methods used to compare groups for primary and secondary outcomes | 10 | | |  |
|  | 12b | Methods for additional analyses, such as subgroup analyses and adjusted analyses | 10 | | |  |
| Results | | | | |  |  |
| Participant flow (a diagram is strongly recommended) | 13a | For each group, the numbers of participants who were randomly assigned, received intended treatment, and were analysed for the primary outcome | , Fig 1 | | |  |
|  | 13b | For each group, losses and exclusions after randomisation, together with reasons | 8, Fig 1 | | |  |
| Recruitment | 14a | Dates defining the periods of recruitment and follow-up | 7 | | |  |
|  | 14b | Why the trial ended or was stopped | Nil | | |  |
| Baseline data | 15 | A table showing baseline demographic and clinical characteristics for each group | 12-13, Table 1 | | |  |
| Numbers analysed | 16 | For each group, number of participants (denominator) included in each analysis and whether the analysis was by original assigned groups | 15-16 | | |  |
| Outcomes and estimation | 17a | For each primary and secondary outcome, results for each group, and the estimated effect size and its precision (such as 95% confidence interval) | 15-16 | | |  |
|  | 17b | For binary outcomes, presentation of both absolute and relative effect sizes is recommended | Nil | | |  |
| Ancillary analyses | 18 | Results of any other analyses performed, including subgroup analyses and adjusted analyses, distinguishing pre-specified from exploratory | Nil | | |  |
| Harms | 19 | All important harms or unintended effects in each group (for specific guidance see CONSORT for harms) | Nil | | |  |
| Discussion | | | | |  |  |
| Limitations | 20 | Trial limitations, addressing sources of potential bias, imprecision, and, if relevant, multiplicity of analyses | 24 | | |  |
| Generalisability | 21 | Generalisability (external validity, applicability) of the trial findings | 24 | | |  |
| Interpretation | 22 | Interpretation consistent with results, balancing benefits and harms, and considering other relevant evidence | 24-25 | | |  |
| Other information | | | |  | | |
| Registration | 23 | Registration number and name of trial registry | 5 | | |  |
| Protocol | 24 | Where the full trial protocol can be accessed, if available | 5 (Ref 26) | | |  |
| Funding | 25 | Sources of funding and other support (such as supply of drugs), role of funders | 26 | | |  |

References:

Schulz KF, Altman DG, Moher D; CONSORT Group. CONSORT 2010 statement: updated guidelines for reporting parallel group randomized trials. Ann Intern Med. 2010;152(11):726–32.

Moher D, Hopewell S, Schulz KF, Montori V, Gøtzsche PC, Devereaux PJ, et al. CONSORT 2010 explanation and elaboration: updated guidelines for reporting parallel group randomised trials. BMJ. 2010;340:c869.
