# Supplementary material for: Effectiveness of Routine Measurement of Health-Related Quality of Life (HRQOL) in Improving Patient-reported Outcomes in Primary Care Patients with Chronic Knee and Back Problems – A Cluster Randomised Controlled Trial
Source: PLOS Digit Health. 2026 Apr 15;5(4):e0001337. doi: 10.1371/journal.pdig.0001337 (PMC13082660; doi:10.1371/journal.pdig.0001337)
Supplement: S1 Table — (DOCX) [file pdig.0001337.s003.docx]

**S1 Table.** **Comparison of baseline characteristics between participants who had completed the study and participants who had defaulted follow up (N = 1328)**

|  | |  | Study participants (N= 1200) | Participants lost to follow up  (N= 128) |  | P-value |  |
| --- | --- | --- | --- | --- | --- | --- | --- |
|  |  |  | n (%)/ mean (SD) | |  |  |  |
| **Socio-Demographic** | |  |  |  |  |  |  |
| Sex | |  |  |  |  | 0.272 |  |
| Male | |  | 371 (30.9) | 33 (25.8) |  |  |  |
| Female | |  | 829 (69.1) | 95 (74.2) |  |  |  |
| Age (years old) | |  | 68.80 (10.24) | 66.73 (10.96) |  | 0.032 |  |
| Education | |  |  |  |  | <0.001 |  |
| Primary or less | |  | 635 (52.9) | 15 (13.4) |  |  |  |
| Secondary | |  | 463 (38.6) | 37 (33.0) |  |  |  |
| Tertiary or above | |  | 100 (8.3) | 60 (53.6) |  |  |  |
| Marital status | |  |  |  |  | 0.766 |  |
| Never married | |  | 77 (6.4) | 10 (7.9) |  |  |  |
| Married | |  | 923 (76.9) | 93 (73.8) |  |  |  |
| Separated/ divorced | |  | 43 (3.6) | 4 (3.2) |  |  |  |
| Widowed | |  | 151 (12.6) | 19 (15.1) |  |  |  |
| Occupation | |  |  |  |  | 0.025 |  |
| Unemployed/Retired | |  | 627 (52.3) | 72 (59.0) |  |  |  |
| Homemaker | |  | 282 (23.5) | 23 (18.9) |  |  |  |
| Labour worker | |  | 126 (10.5) | 13 (10.7) |  |  |  |
| Clerical Worker | |  | 48 (4.0) | 9 (7.4) |  |  |  |
| Professional/Manager | |  | 27 (2.3) | 5 (4.1) |  |  |  |
| Others | |  | 67 (5.6) | 0 (0.0) |  |  |  |
| Household monthly income (HKD) ^†^ | | |  |  |  | 0.005 |  |
| 0-$9999 | |  | 604 (50.3) | 47 (47.5) |  |  |  |
| $10000-19999 | |  | 156 (13.0) | 18 (18.2) |  |  |  |
| $20000-29999 | |  | 76 (6.3) | 13 (13.1) |  |  |  |
| >$29999 | |  | 112 (9.3) | 21 (21.2) |  |  |  |
| Smoking | |  |  |  |  | 0.231 |  |
| Non-smoker | |  | 1068 (89.0) | 119 (93.0) |  |  |  |
| Ex-smoker | |  | 85 (7.1) | 4 (3.1) |  |  |  |
| Current Smoker | |  | 43 (3.6) | 5 (3.9) |  |  |  |
| Alcohol drinking | |  |  |  |  | 0.042 |  |
| Non-drinker | |  | 1034 (86.2) | 111 (86.7) |  |  |  |
| Ex-drinker | |  | 46 (3.8) | 0 (0.0) |  |  |  |
| Current drinker ^§^ | |  | 117 (9.8) | 17 (13.3) |  |  |  |
| **Disease characteristics** | |  |  |  |  |  |  |
|  | Diagnosis of musculoskeletal problem | | |  |  | <0.001 |  |
| Back only | |  | 266 (22.2) | 55 (44.0) |  |  |  |
| Knee only | |  | 814 (67.8) | 44 (35.2) |  |  |  |
| Both | |  | 120 (10.0) | 26 (20.8) |  |  |  |
| Duration | |  |  |  |  | <0.001 |  |
| <1 year | |  | 201 (16.8) | 33 (25.8) |  |  |  |
| 1-5 years | |  | 398 (33.2) | 39 (30.5) |  |  |  |
| 5-10 years | |  | 223 (18.6) | 7 (5.5) |  |  |  |
| >10 years | |  | 360 (30.0) | 49 (38.3) |  |  |  |
| Total number of comorbidities | | | 1.39 (±0.95) | 1.28 (1.06) |  | 0.307 |  |
| Comorbidities | |  |  |  |  |  |  |
| No chronic disease | |  | 107 (8.9) | 18 (14.1) |  | 0.034 |  |
| Heart disease | |  | 100 (8.3) | 11 (8.6) |  | 1 |  |
| Hypertension | |  | 900 (75.0) | 79 (61.7) |  | 0.002 |  |
| Stroke | |  | 37 (3.1) | 6 (4.7) |  | 0.476 |  |
| Diabetes | |  | 324 (27.0) | 23 (18.0) |  | 0.035 |  |
| Lung disease | |  | 27 (2.3) | 3 (2.3) |  | 1 |  |
| Mental illness | |  | 50 (4.2) | 8 (6.2) |  | 0.385 |  |
| Kidney disease | |  | 17 (1.4) | 5 (3.9) |  | 0.083 |  |
| Other joint problem | |  | 162 (13.5) | 26 (20.3) |  | 0.049 |  |
| Cancer | |  | 24 (2.0) | 3 (2.3) |  | 1 |  |
| Other diseases | |  | 188 (15.7) | 23 (18.0) |  | 0.582 |  |
| WOMAC total score (range 0 to 96) | |  | 20.62±14.42 | 22.53 (15.20) |  | 0.157 |  |
| WOMAC pain score (range 0 to 20) | |  | 5.12±3.44 | 5.60 (3.70) |  | 0.132 |  |
| WOMAC stiffness score (range 0 to 8) | |  | 1.62±1.67 | 1.85 (1.63) |  | 0.139 |  |
| WOMAC function score (range 0 to 68) | |  | 13.88±10.77 | 15.07 (11.53) |  | 0.236 |  |
| PEI-2 total score (range 6 to 30) | |  | 21.61±3.44 | 21.02 (4.05) |  | 0.069 |  |
| SF-6D utility score (range 0.291 to 1) | |  | 0.72±0.15 | 0.74 (0.10) |  | 0.070 |  |
| PRS score (range 0 to 10) | |  | 5.40±2.30 | 5.32 (2.21) |  | 0.728 |  |
|  | **Prevalence of self-reported treatment/healthcare service utilized in the 3 months prior to start of study** | | | | | |  |
| Oral prescribed medication | |  | 657 (54.8) | 76 (59.4) |  | 0.380 |  |
| Physiotherapy | |  | 121 (10.1) | 27 (21.1) |  | <0.001 |  |
| Occupational therapy | |  | 11 (0.9) | 4 (3.1) |  | 0.071 |  |
| Surgery | |  | 26 (2.2) | 9 (7.0) |  | 0.003 |  |
| Sick leaves | |  | 28 (2.3) | 7 (5.5) |  | 0.07 |  |
| Self-medication | |  | 308 (25.7) | 42 (32.8) |  | 0.106 |  |
| Accident & Emergency visits | |  | 15 (1.3) | 3 (2.3) |  | 0.54 |  |
| Specialist outpatient visits | |  | 83 (6.9) | 18 (14.1) |  | 0.007 |  |
| Hospital admission | |  | 17 (1.4) | 4 (3.1) |  | 0.273 |  |
