# Supplementary material for: Effectiveness of Routine Measurement of Health-Related Quality of Life (HRQOL) in Improving Patient-reported Outcomes in Primary Care Patients with Chronic Knee and Back Problems – A Cluster Randomised Controlled Trial
Source: PLOS Digit Health. 2026 Apr 15;5(4):e0001337. doi: 10.1371/journal.pdig.0001337 (PMC13082660; doi:10.1371/journal.pdig.0001337)
Supplement: S2 Table — (DOCX) [file pdig.0001337.s004.docx]

# **S2 Table. Baseline characteristics of study participants by clinics (N = 1200)**

|  | | | | | All participants (N= 1200) | | Clinic 1 (n=232) | | | Clinic 2 (n=114) | | Clinic 3 (n=242) | | Clinic 4 (n=171) | | Clinic 5 (n=192) | | Clinic 6 (n=249) |  | Intraclass correlation coefficient (ICC) |  |
| --- | --- | --- | --- | --- | --- | --- | --- | --- | --- | --- | --- | --- | --- | --- | --- | --- | --- | --- | --- | --- | --- |
|  |  |  |  |  |  |  | n (%)/ mean (SD) | | | | | | | | | | | |  |  |  |
| **Socio-Demographic** | | | | |  | |  | | |  | |  | |  | |  | |  |  |  |  |
| Sex | | | | |  | |  | | |  | |  | |  | |  | |  |  | 0.05 |  |
| Male | | | | | 371 (30.9) | | 68 (29.3) | | | 41 (36.0) | | 63 (26.0) | | 49 (28.7) | | 62 (32.3) | | 88 (35.3) |  |  |  |
| Female | | | | | 829 (69.1) | | 164 (70.7) | | | 73 (64.0) | | 179 (74.0) | | 122 (71.3) | | 130 (67.7) | | 161 (64.7) |  |  |  |
| Age (years old) | | | | | 68.80±10.24 | | 66.45 (10.11) | | | 71.59 (11.07) | | 67.68 (10.54) | | 68.75 (11.01) | | 70.43 (9.46) | | 69.56 (9.14) |  | 0.03 |  |
| Education | | | | |  | |  | | |  | |  | |  | |  | |  |  | 0.01 |  |
| Primary or less | | | | | 635 (52.9) | | 135 (58.2) | | | 58 (50.9) | | 105 (43.8) | | 88 (51.5) | | 124 (64.6) | | 125 (50.2) |  |  |  |
| Secondary | | | | | 463 (38.6) | | 78 (33.6) | | | 46 (40.4) | | 99 (41.2) | | 72 (42.1) | | 58 (30.2) | | 110 (44.2) |  |  |  |
| Tertiary or above | | | | | 100 (8.3) | | 19 (8.2) | | | 10 (8.8) | | 36 (15.0) | | 11 (6.4) | | 10 (5.2) | | 14 (5.6) |  |  |  |
| Marital status | | | | |  | |  | | |  | |  | |  | |  | |  |  | 0.01 |  |
| Never married | | | | | 77 (6.4) | | 18 (7.8) | | | 9 (7.9) | | 20 (8.4) | | 12 (7.0) | | 11 (5.7) | | 7 (2.8) |  |  |  |
| Married | | | | | 923 (76.9) | | 177 (77.0) | | | 93 (81.6) | | 164 (68.9) | | 136 (79.5) | | 127 (66.1) | | 226 (90.8) |  |  |  |
| Separated/ divorced | | | | | 43 (3.6) | | 8 (3.5) | | | 3 (2.6) | | 12 (5.0) | | 6 (3.5) | | 10 (5.2) | | 4 (1.6) |  |  |  |
| Widowed | | | | | 151 (12.6) | | 27 (11.7) | | | 9 (7.9) | | 42 (17.6) | | 17 (9.9) | | 44 (22.9) | | 12 (4.8) |  |  |  |
| Occupation | | | | |  | |  | | |  | |  | |  | |  | |  |  | 0.02 |  |
| Unemployed/Retired | | | | | 627 (52.3) | | 95 (40.9) | | | 88 (77.2) | | 118 (49.4) | | 131 (76.6) | | 48 (25.0) | | 167 (67.1) |  |  |  |
| Homemaker | | | | | 282 (23.5) | | 71 (30.6) | | | 4 (3.5) | | 61 (25.5) | | 2 (1.2) | | 118 (61.5) | | 26 (10.4) |  |  |  |
| Labour worker | | | | | 126 (10.5) | | 26 (11.2) | | | 18 (15.8) | | 22 (9.2) | | 20 (11.7) | | 4 (2.1) | | 36 (14.5) |  |  |  |
| Clerical Worker | | | | | 48 (4.0) | | 12 (5.2) | | | 3 (2.6) | | 15 (6.3) | | 7 (4.1) | | 2 (1.0) | | 9 (3.6) |  |  |  |
| Professional/Manager | | | | | 27 (2.3) | | 7 (3.0) | | | 0 (0.0) | | 2 (0.8) | | 8 (4.7) | | 1 (0.5) | | 9 (3.6) |  |  |  |
| Others | | | | | 67 (5.6) | | 21 (9.1) | | | 1 (0.9) | | 21 (8.8) | | 3 (1.8) | | 19 (9.9) | | 2 (0.8) |  |  |  |
| Household monthly income (HKD) ^†^ | | | | | | |  | | |  | |  | |  | |  | |  |  | 0.02 |  |
| 0-$9999 | | | | | 604 (50.3) | | 78 (45.9) | | | 65 (67.0) | | 101 (53.4) | | 115 (78.8) | | 81 (65.9) | | 164 (73.5) |  |  |  |
| $10000-19999 | | | | | 156 (13.0) | | 36 (21.2) | | | 23 (23.7) | | 27 (14.3) | | 19 (13.0) | | 16 (13.0) | | 35 (15.7) |  |  |  |
| $20000-29999 | | | | | 76 (6.3) | | 25 (14.7) | | | 3 (3.1) | | 19 (10.1) | | 4 (2.7) | | 14 (11.4) | | 11 (4.9) |  |  |  |
| >$29999 | | | | | 112 (9.3) | | 31 (18.2) | | | 6 (6.2) | | 42 (22.2) | | 8 (5.5) | | 12 (9.8) | | 13 (5.8) |  |  |  |
| Smoking | | | | |  | |  | | |  | |  | |  | |  | |  |  | 0.01 |  |
| Non-smoker | | | | | 1068 (89.0) | | 220 (94.8) | | | 100 (88.5) | | 217 (90.4) | | 152 (88.9) | | 161 (84.3) | | 218 (87.6) |  |  |  |
| Ex-smoker | | | | | 85 (7.1) | | 7 (3.0) | | | 7 (6.2) | | 17 (7.1) | | 8 (4.7) | | 26 (13.6) | | 20 (8.0) |  |  |  |
| Current Smoker | | | | | 43 (3.6) | | 5 (2.2) | | | 6 (5.3) | | 6 (2.5) | | 11 (6.4) | | 4 (2.1) | | 11 (4.4) |  |  |  |
| Alcohol drinking | | | | |  | |  | | |  | |  | |  | |  | |  |  | 0.03 |  |
| Non-drinker | | | | | 1034 (86.2) | | 195 (84.1) | | | 98 (86.7) | | 207 (86.2) | | 157 (91.8) | | 156 (81.2) | | 221 (88.8) |  |  |  |
| Ex-drinker | | | | | 46 (3.8) | | 5 (2.2) | | | 2 (1.8) | | 8 (3.3) | | 4 (2.3) | | 19 (9.9) | | 8 (3.2) |  |  |  |
| Current drinker ^§^ | | | | | 117 (9.8) | | 32 (13.8) | | | 13 (11.5) | | 25 (10.4) | | 10 (5.8) | | 17 (8.9) | | 20 (8.0) |  |  |  |
| **Disease characteristics** | | | | |  | |  | | |  | |  | |  | |  | |  |  |  |  |
|  | Diagnosis of musculoskeletal problem | | |  | | | |  | | |  | | | |  | |  | |  | 0.01 |  |
| Back only | | | | | 266 (22.2) | | 41 (17.7) | | | 22 (19.3) | | 49 (20.2) | | 58 (33.9) | | 36 (18.8) | | 60 (24.1) |  |  |  |
| Knee only | | | | | 814 (67.8) | | 173 (74.6) | | | 87 (76.3) | | 160 (66.1) | | 82 (48.0) | | 137 (71.4) | | 175 (70.3) |  |  |  |
| Both | | | | | 120 (10.0) | | 18 (7.8) | | | 5 (4.4) | | 33 (13.6) | | 31 (18.1) | | 19 (9.9) | | 14 (5.6) |  |  |  |
| Duration | | | | |  | |  | | |  | |  | |  | |  | |  |  | 0.01 |  |
| <1 year | | | | | 201 (16.8) | | 44 (19.2) | | | 27 (23.7) | | 48 (20.3) | | 9 (5.4) | | 41 (21.4) | | 32 (13.2) |  |  |  |
| 1-5 years | | | | | 398 (33.2) | | 85 (37.1) | | | 41 (36.0) | | 76 (32.1) | | 52 (31.0) | | 73 (38.0) | | 71 (29.3) |  |  |  |
| 5-10 years | | | | | 223 (18.6) | | 34 (14.8) | | | 15 (13.2) | | 38 (16.0) | | 52 (31.0) | | 31 (16.1) | | 53 (21.9) |  |  |  |
| >10 years | | | | | 360 (30.0) | | 66 (28.8) | | | 31 (27.2) | | 75 (31.6) | | 55 (32.7) | | 47 (24.5) | | 86 (35.5) |  |  |  |
| Total number of comorbidities | | | | | | | 1.36 (1.02) | | | 1.23 (0.70) | | 1.42 (1.02) | | 1.42 (0.97) | | 1.40 (0.85) | | 1.33 (0.62) |  | 0.01 |  |
| Comorbidities | | | | |  | |  | | |  | |  | |  | |  | |  |  |  |  |
| No chronic disease | | | | | 107 (8.9) | | 25 (10.8) | | | 5 (4.4) | | 22 (9.1) | | 30 (17.5) | | 12 (6.2) | | 3 (1.2) |  | 0.04 |  |
| Heart disease | | | | | 100 (8.3) | | 15 (6.5) | | | 6 (5.3) | | 21 (8.7) | | 32 (18.7) | | 8 (4.2) | | 18 (7.2) |  | 0.01 |  |
| Hypertension | | | | | 900 (75.0) | | 165 (71.1) | | | 84 (73.7) | | 168 (69.4) | | 100 (58.5) | | 162 (84.4) | | 221 (88.8) |  | 0.02 |  |
| Stroke | | | | | 37 (3.1) | | 6 (2.6) | | | 3 (2.6) | | 6 (2.5) | | 11 (6.4) | | 4 (2.1) | | 7 (2.8) |  | 0.02 |  |
| Diabetes | | | | | 324 (27.0) | | 66 (28.4) | | | 29 (25.4) | | 46 (19.0) | | 64 (37.4) | | 50 (26.0) | | 69 (27.7) |  | 0.03 |  |
| Lung disease | | | | | 27 (2.3) | | 5 (2.2) | | | 0 (0.0) | | 8 (3.3) | | 4 (2.3) | | 9 (4.7) | | 1 (0.4) |  | 0.02 |  |
| Mental illness | | | | | 50 (4.2) | | 12 (5.2) | | | 7 (6.1) | | 14 (5.8) | | 2 (1.2) | | 10 (5.2) | | 5 (2.0) |  | 0.05 |  |
| Kidney disease | | | | | 17 (1.4) | | 8 (3.4) | | | 0 (0.0) | | 3 (1.2) | | 1 (0.6) | | 3 (1.6) | | 2 (0.8) |  | 0.03 |  |
| Other joint problem | | | | | 162 (13.5) | | 31 (13.4) | | | 10 (8.8) | | 72 (29.8) | | 25 (14.6) | | 16 (8.3) | | 8 (3.2) |  | 0.02 |  |
| Cancer | | | | | 24 (2.0) | | 7 (3.0) | | | 1 (0.9) | | 6 (2.5) | | 3 (1.8) | | 6 (3.1) | | 1 (0.4) |  | 0.02 |  |
| Other diseases | | | | | 188 (15.7) | | 20 (8.6) | | | 17 (14.9) | | 40 (16.5) | | 25 (14.6) | | 16 (8.3) | | 70 (28.1) |  | 0.05 |  |
| Doctor-reported GRS score (range 0-4) | | | | | 2.44±0.64 | | 1.55 (0.70) | | | 1.45 (0.68) | | 1.42 (0.65) | | 1.45 (0.65) | | 1.42 (0.60) | | 1.37 (0.57) |  | 0.01 |  |
| WOMAC total score (range 0 to 96) | | | | | 20.62±14.42 | | 19.05 (14.62) | | | 19.65 (15.86) | | 23.54 (15.84) | | 22.22 (12.88) | | 19.48 (13.54) | | 19.45 (13.33) |  | 0.01 |  |
| WOMAC pain score (range 0 to 20) | | | | | 5.12±3.44 | | 4.79 (3.47) | | | 4.97 (4.03) | | 5.52 (3.62) | | 5.31 (3.16) | | 4.93 (3.28) | | 5.10 (3.25) |  | 0.01 |  |
| WOMAC stiffness score (range 0 to 8) | | | | | 1.62±1.67 | | 1.55 (1.61) | | | 1.23 (1.54) | | 1.92 (1.76) | | 1.79 (1.60) | | 1.43 (1.59) | | 1.60 (1.73) |  | 0.02 |  |
| WOMAC function score (range 0 to 68) | | | | | 13.88±10.77 | | 12.71 (10.82) | | | 13.44 (11.64) | | 16.10 (11.98) | | 15.12 (9.62) | | 13.11 (9.88) | | 12.75 (10.10) |  | 0.01 |  |
| PEI-2 total score (range 6 to 30) | | | | | 21.61±3.44 | | 22.02 (3.71) | | | 22.89 (5.05) | | 21.50 (3.22) | | 20.80 (3.10) | | 22.01 (2.16) | | 21.01 (3.28) |  | 0.04 |  |
| SF-6D utility score (range 0.291 to 1) | | | | | 0.72±0.15 | | 0.74 (0.15) | | | 0.70 (0.15) | | 0.69 (0.15) | | 0.68 (0.14) | | 0.74 (0.16) | | 0.73 (0.12) |  | 0.02 |  |
| PRS score (range 0 to 10) | | | | | 5.40±2.30 | | 5.17 (2.52) | | | 5.17 (2.23) | | 5.19 (2.23) | | 5.71 (1.83) | | 5.68 (2.74) | | 5.49 (2.06) |  | 0.01 |  |
| **Prevalence of self-reported treatment/healthcare service utilized in the 3 months prior to start of study** | | | | | | | | | | | | | | | | | | | | |  |
| Oral prescribed medication | | | | | 657 (54.8) | | 138 (59.7) | | | 54 (47.4) | | 135 (56.0) | | 99 (58.2) | | 111 (57.8) | | 120 (48.2) |  | 0.04 |  |
| Physiotherapy | | | | | 121 (10.1) | | 16 (6.9) | | | 12 (10.5) | | 30 (12.4) | | 17 (9.9) | | 19 (9.9) | | 27 (10.9) |  | 0.02 |  |
| Occupational therapy | | | | | 11 (0.9) | | 1 (0.4) | | | 1 (0.9) | | 4 (1.7) | | 1 (0.6) | | 2 (1.0) | | 2 (0.8) |  | 0.01 |  |
| Surgery | | | | | 26 (2.2) | | 6 (2.6) | | | 1 (0.9) | | 13 (5.4) | | 0 (0.0) | | 3 (1.6) | | 3 (1.2) |  | 0.09 |  |
| Sick leaves | | | | | 28 (2.3) | | 7 (3.0) | | | 1 (0.9) | | 6 (2.5) | | 4 (2.3) | | 3 (1.6) | | 7 (2.8) |  | 0.02 |  |
| Self-medication | | | | | 308 (25.7) | | 61 (26.5) | | | 16 (14.0) | | 65 (27.0) | | 17 (9.9) | | 35 (18.2) | | 114 (46.0) |  | 0.05 |  |
| Accident & Emergency visits | | | | | 15 (1.3) | | 2 (0.9) | | | 0 (0.0) | | 5 (2.1) | | 0 (0.0) | | 5 (2.6) | | 3 (1.2) |  | 0.01 |  |
| Specialist outpatient visits | | | | | 83 (6.9) | | 20 (8.7) | | | 5 (4.4) | | 22 (9.1) | | 4 (2.3) | | 14 (7.3) | | 18 (7.2) |  | 0.03 |  |
| Hospital admission | | | | | 17 (1.4) | | 1 (0.4) | | | 2 (1.8) | | 5 (2.1) | | 1 (0.6) | | 6 (3.1) | | 2 (0.8) |  | 0.03 |  |
|  |  |  |  | | |  | | |  | | | |  |  |  |  |  |  |  |  |  |
