# Supplementary material for: Effectiveness of Routine Measurement of Health-Related Quality of Life (HRQOL) in Improving Patient-reported Outcomes in Primary Care Patients with Chronic Knee and Back Problems – A Cluster Randomised Controlled Trial
Source: PLOS Digit Health. 2026 Apr 15;5(4):e0001337. doi: 10.1371/journal.pdig.0001337 (PMC13082660; doi:10.1371/journal.pdig.0001337)
Supplement: S3 Table — (DOCX) [file pdig.0001337.s005.docx]

| **S3 Table. Effects of intervention by routine e-EQ5D5L measurement on changes in patient-reported outcomes from baseline to 3, 6 and 12-month follow-up by GEE with cluster robust standard errors (N=1200)** | | | | | | | | | | | | | | | | |
| --- | --- | --- | --- | --- | --- | --- | --- | --- | --- | --- | --- | --- | --- | --- | --- | --- |
| Patient-reported outcomes | WOMAC total score (n= 1198) | | | | SF6D utility score (n= 1194) | | | | PEI-2 total score (n= 1189) | | | | PRS (n =1199) | | | |
|  | Unadjusted | | Adjusted | | Unadjusted | | Adjusted | | Unadjusted | | Adjusted | | Unadjusted | | Adjusted | |
|  | β  (95 CI) | p- value | β^§^  (95 CI) | p- value | β  (95 CI) | p- value | β^§^  (95 CI) | p- value | β  (95 CI) | p- value | β^§^  (95 CI) | p- value | β  (95 CI) | p- value | β^§^  (95 CI) | p- value |
| **Time (vs Baseline) * Intervention (vs Control)** | | | | | | | | | | | | | | | | |
| 12 months | 2.69  (1.04, 4.33) | 0.001* | 2.65  (0.59, 4.71 | 0.017* | -0.01  (-0.04, 0.01) | 0.308 | -0.01  (-0.05, 0.02) | 0.388 | 0.83  (-0.40, 2.06) | 0.184 | 0.98  (-0.36, 2.32) | 0.152 | 0.10  (-0.36, 0.56) | 0.669 | 0.09  (-0.35, 0.54) | 0.688 |
| 6 months | 1.48  (-0.78, 3.73) | 0.198 | 1.39  (-1.19, 3.97) | 0.290 | -0.03  (-0.05,  -0.01) | 0.003* | -0.03  (-0.06,  -0.01) | 0.001* | -0.23  (-1.33, 0.87) | 0.683 | -0.04  (-1.21, 1.12) | 0.941 | -0.06  (-0.53,  0.43) | 0.821 | -0.12  (-0.59, 0.36) | 0.631 |
| 3 months | 1.80  (-2.32, 5.92) | 0.392 | 1.35  (-2.66, 5.36) | 0.510 | -0.03  (-0.07,  0.01) | 0.103 | -0.03  (-0.06,  -0.01) | 0.015 | -0.29  (-1.23, 0.64) | 0.541 | -0.14  (-1.16,  0.87) | 0.786 | -0.01  (-0.23,  0.23) | <0.001* | -0.09  (-0.41,  0.22) | 0.560 |
| e-EQ5D5L= electronic-EuroQol 5-Dimension 5-Level; PEI-2= Patient Enablement Instrument-version 2 (higher score indicates more enabled); PRS= Pain Rating Scale (higher score indicates more pain); SD = standard deviation; SF-6D=Short-Form Six-Dimension (higher score indicates higher health utility); WOMAC= The Western Ontario and McMaster Universities Osteoarthritis Index (higher score indicates more limitation); 95 CI = 95% of the confidence interval.  **Notes**.  Analysis by a generalised estimating equation (GEE) with a linear function, an exchangeable working correlation structure, and cluster robust standard errors to account for correlation of observations within clusters.  § Models were adjusted for age, gender, baseline outcome score, baseline global rating of severity, diagnosis type, duration of diagnosis, and number of comorbidities.  * Significant at p<0.05. | | | | | | | | | | | | | | | | |
