# Supplementary material for: Effectiveness of Routine Measurement of Health-Related Quality of Life (HRQOL) in Improving Patient-reported Outcomes in Primary Care Patients with Chronic Knee and Back Problems – A Cluster Randomised Controlled Trial
Source: PLOS Digit Health. 2026 Apr 15;5(4):e0001337. doi: 10.1371/journal.pdig.0001337 (PMC13082660; doi:10.1371/journal.pdig.0001337)
Supplement: S4 Table — (DOCX) [file pdig.0001337.s006.docx]

# **S4 Table. Prevalence of patient-reported treatment and service utilization throughout the study period by groups (N=1200)**

|  | Intervention (N=595) | Control  (N=605) | Odds Ratio (95 CI) | p-value | Adjusted  Odds Ratio^§^ (95 CI) | p-value |
| --- | --- | --- | --- | --- | --- | --- |
|  | n (%) | |  |  |  |  |
| **Prevalence of receiving treatment within the study period** | |  |  |  |  |  |
| Topical medication | 457 (81.8) | 415 (68.6) | 1.516 (1.173,1.959) | 0.001 | 1.479 (1.139,1.922) | 0.003 |
| Oral medication | 443 (79.2) | 367 (60.7) | 1.890 (1.478,2.417) | <0.001 | 1.807 (1.402, 2.331) | <0.001 |
| Injection medication | 36 (6.4) | 35 (5.8) | 1.049 (0.649, 1.694) | 0.846 | 1.103 (0.672, 1.810) | 0.699 |
| Physiotherapy | 176 (31.5) | 189 (31.2) | 0.925 (0.723,1.182) | 0.532 | 0.895 (0.691, 1.158) | 0.398 |
| Occupational therapy | 35 (6.3) | 29 (4.8) | 1.241 (0.749,2.058) | 0.402 | 1.108 (0.661, 1.858) | 0.696 |
| Local injection | 12 (2.1) | 17 (2.8) | 0.712 (0.337,1.504) | 0.373 | 0.777 (0.356, 1.696) | 0.526 |
| Surgery | 543 (97.1) | 551 (91.1) | 1.023 (0.687,1.525) | 0.910 | 1.039 (0.695, 1.555) | 0.850 |
| Psychological consultation | 12 (2.1) | 12 (2.0) | 1.017 (0.453,2.283) | 0.967 | 0.934 (0.411, 2.121) | 0.870 |
| **Prevalence of MSK-related service utilization within the study period** | |  |  |  |  |  |
| Sick leaves | 20 (3.6) | 17 (2.8) | 1.203 (0.624,2.320) | 0.581 | 1.055 (0.526, 2.114) | 0.881 |
| Western doctor visits | 134 (24.0) | 144 (23.8) | 0.931 (0.712,1.217) | 0.599 | 0.879 (0.664, 1.163) | 0.367 |
| Chinese doctor visits | 110 (19.7) | 99 (16.4) | 1.159 (0.860, 1.563) | 0.332 | 1.122 (0.825, 1.525) | 0.464 |
| Self-medication | 185 (33.1) | 162 (26.8) | 1.234 (0.961,1.584) | 0.099 | 1.170 (0.906, 1.511) | 0.230 |
| Accident & Emergency visits | 18 (3.2) | 20 (3.3) | 0.912 (0.478,1.743) | 0.781 | 0.967 (0.496, 1.884) | 0.921 |
| Specialist outpatient visits | 86 (15.4) | 103 (17.0) | 0.823 (0.603,1.125) | 0.222 | 0.739 (0.531, 1.028) | 0.072 |
| Hospital admission | 16 (2.9) | 28 (4.6) | 0.758 (0.355,1.616) | 0.473 | 0.732 (0.340, 1.576) | 0.425 |
| **Non-MSK-related service utilization within the study period** | |  |  |  |  |  |
| Sick leaves | 37 (6.6) | 23 (3.8) | 1.678 (0.984,2.860) | 0.057 | 1.653 (0.938, 2.915) | 0.082 |
| Western doctor visits | 245 (43.8) | 275 (45.5) | 0.840 (0.668,1.056) | 0.135 | 0.844 (0.666, 1.069) | 0.160 |
| Chinese doctor visits | 83 (14.8) | 78 (12.9) | 1.095 (0.786,1.527) | 0.591 | 1.087 (0.773, 1.527) | 0.632 |
| Self-medication | 106 (19.0) | 100 (16.5) | 1.095 (0.811,1.478) | 0.555 | 1.117 (0.822, 1.517) | 0.480 |
| Accident & Emergency visits | 30 (5.4) | 30 (5.0) | 1.018 (0.606,1.710) | 0.947 | 1.091 (0.630, 1.889) | 0.757 |
| Specialist outpatient visits | 131 (23.4) | 167 (27.6) | 0.740 (0.569,0.964) | 0.025 | 0.716 (0.545, 0.940) | 0.016 |
| Hospital admission | 41 (7.3) | 29 (4.8) | 1.470 (0.901,2.399) | 0.123 | 1.502 (0.915, 2.463) | 0.107 |

Note. Logistic regression was used for analyses. Odds Ratio = odds in intervention group divided by odds in control group; ^§^Adjusted by age, gender, global rating severity of baseline, diagnosed type, diagnosed duration, and number of comorbidities.
