# Supplementary material for: Effectiveness of Routine Measurement of Health-Related Quality of Life (HRQOL) in Improving Patient-reported Outcomes in Primary Care Patients with Chronic Knee and Back Problems – A Cluster Randomised Controlled Trial
Source: PLOS Digit Health. 2026 Apr 15;5(4):e0001337. doi: 10.1371/journal.pdig.0001337 (PMC13082660; doi:10.1371/journal.pdig.0001337)
Supplement: Attachment — Submitted filename: PLOS One digital health.docx [file pdig.0001337.s007.docx]

**PLOS One digital health**

**PDIG-D-25-00766
Effectiveness of Routine Measurement of Health-Related Quality of Life (HRQOL) in Improving Patient-reported Outcomes in Primary Care Patients with Chronic Knee and Back Problems – A Cluster Randomised Controlled Trial**

I would like to thank the editorial team for giving me the opportunity to review this manuscript. Also appreciate the authors’ efforts in addressing an important area of clinical research related to improving patient-reported outcomes in primary care. The topic is both timely and relevant, considering the growing emphasis on integrating HRQOL measures into chronic disease management.

**Abstract**

1. The abstract clearly presents the study design and findings; however, the **background section** could be strengthened by briefly explaining *why* measuring HRQOL routinely is expected to improve patient outcomes.

2. The **conclusion** is informative but slightly overstated. It should reflect that HRQOL measurement did not improve HRQOL or pain, but was linked to improved enablement

**Introduction**

3. The introduction provides a clear and logical background, but it could be strengthened by explicitly highlighting the knowledge gap i.e., although HRQOL measures are known to enhance patient–doctor communication, their actual clinical impact on outcomes in primary care remains uncertain

4. The definition of patient enablement is appropriate; still, linking it more explicitly to HRQOL improvement and chronic disease self-management would make the argument more cohesive.

5. Minor language refinements e.g., “to facilitate routine HRQOL measurement in busy primary care clinics, our team developed…” instead of “to facilitate routine HRQOL measurement in busy primary care clinics our research team has developed” would improve readability.

**Method**

6. The study design is appropriate (cluster RCT, 12 months, single-blind), but it would be useful to explain *why* the cluster design was chosen and whether intra-cluster correlation was accounted for in the sample size calculation and analysis.

7. Please clarify what “single-blind” refers to participants, doctors, or outcome assessors

8. The target sample size (n=1374) is reported, but the **sample size calculation formula and assumptions** (effect size, power, ICC, etc.) are not shown here.

9. Randomisation at the clinic level is appropriate, but additional detail on allocation concealment and steps taken to minimize selection bias before recruitment would be helpful.

10. Since only six clinics were included, please discuss whether stratification or matching was performed to balance clinic characteristics between groups.

11. Please clarify whether doctors in the intervention group received training or guidance on how to interpret and use HRQOL reports during consultations.

12. The schedule for baseline and follow-up assessments is well defined, but consider clarifying whether the same interviewer conducted all follow-ups and whether inter-rater reliability was assessed.

13. The data collection method (telephone vs face-to-face) differs between baseline and follow-ups; discuss any potential mode effect on data consistency.

14. The use of multiple PROMs is appropriate, but the manuscript should explain whether any correction for multiple comparisons was applied to avoid Type I error inflation.

15. Clarify how missing data were handled

16. The authors should state whether analyses were **intention-to-treat (ITT)** or **per-protocol**.

**Results**

17. The loss to follow-up rate is acceptable, but it should be noted whether attrition was statistically compared between groups and if missing data could have introduced bias.

18. The text mentions *no significant difference in PROMs at baseline*, which supports group comparability; however, please specify whether these comparisons were performed using appropriate tests adjusted for clustering.

19. Clarify whether participants with both knee and back problems were analyzed as a combined group or separately.

20. Although the tables are referenced, the narrative could briefly report key numerical values (e.g., mean changes and confidence intervals) rather than relying mainly on p-values.

21. Effect sizes and clinical significance should be discussed, particularly for WOMAC and PEI-2 outcomes.

22. The authors mention using GEE models, but it would be useful to specify whether they accounted for clustering by clinic and adjusted for baseline covariates.

23. The section could be streamlined by avoiding repetitive mentions of significance for each follow-up.

**Discussion**

24. The discussion could be strengthened by more explicitly addressing the clinical relevance of the observed improvements in enablement scores for instance, whether the magnitude of change in PEI-2 is meaningful in real-world practice.

25. While the authors suggest that HRQOL reporting may improve doctor awareness of psychosocial issues, this point could be expanded with reference to behavioral or patient engagement theories e.g., patient activation, shared decision-making. This would enhance the conceptual grounding of the discussion.

26. The limitations section is concise but could be expanded. Specifically, the impact of COVID-19 restrictions on both recruitment and outcomes deserves more emphasis, as it may have introduced systematic bias or affected patient behaviors differently across clusters.

27. The comparison with prior studies could benefit from a deeper discussion on why previous interventions showed improvements in mental health outcomes and why this trial did not translate that effect into HRQOL or pain reduction.

28. Future research directions could be strengthened by suggesting mixed-methods evaluations or cost-effectiveness analyses to determine the broader value of routine HRQOL measurement.

**Conclusion**

29. A closing remark highlighting the potential policy or primary care implications e.g., supporting personalized management plans would enhance the practical significance of the work.
