# Supplementary material for: Effectiveness of Routine Measurement of Health-Related Quality of Life (HRQOL) in Improving Patient-reported Outcomes in Primary Care Patients with Chronic Knee and Back Problems – A Cluster Randomised Controlled Trial
Source: PLOS Digit Health. 2026 Apr 15;5(4):e0001337. doi: 10.1371/journal.pdig.0001337 (PMC13082660; doi:10.1371/journal.pdig.0001337)
Supplement: Attachment — Submitted filename: Response to Reviewers.docx [file pdig.0001337.s009.docx]

4 January 2026

Dear Editors (Jan Louis Adajo Non, Dukyong Yoon, Dr. Leo Anthony Celi) and Reviewers,

We are grateful for the opportunity to submit a revised version of our manuscript (PDIG-D-25-00766) entitled “Effectiveness of Routine Measurement of Health-Related Quality of Life (HRQOL) in Improving Patient-reported Outcomes in Primary Care Patients with Chronic Knee and Back Problems – A Cluster Randomised Controlled Trial.”

We sincerely appreciate the time and effort that you and the reviewers have invested in providing constructive feedback. In response, we have carefully revised the manuscript to incorporate the majority of the suggestions offered. All changes have been highlighted within the text, and we have provided a detailed point-by-point response to the reviewers’ comments in a separate document.

Should further modifications be required to ensure the manuscript meets the standards of PLOS Digital Health, we would be more than willing to make additional revisions. We are thankful for your encouragement and for the continued consideration of our work.

Thank you very much for your attention and support.

Sincerely,

Dr. Amy Pui Pui Ng

Department of Family Medicine and Primary Care, Li Ka Shing Faculty of Medicine, The University of Hong Kong

3/F., 161 Main Street, Ap Lei Chau Clinic, Ap Lei Chau, Hong Kong

Tel. (852) 2290 5098

Fax. (852) 2814 7475

Reviewer 1

| Reviewer Comment | Response |
| --- | --- |
| I would like to thank the editorial team for giving me the opportunity to review this manuscript. Also appreciate the authors’ efforts in addressing an important area of clinical research related to improving patient-reported outcomes in primary care. The topic is both timely and relevant, considering the growing emphasis on integrating HRQOL measures into chronic disease management. | We thank the reviewer for the endorsement. |
| **Abstract**  1. The abstract clearly presents the study design and findings; however, the **background section** could be strengthened by briefly explaining *why* measuring HRQOL routinely is expected to improve patient outcomes. | We have added a sentence on how HRQOL can help improve patient outcomes.  *“Routine HRQOL measurement may enhance doctor-patient communication and person-centred care by identifying unmet biopsychosocial needs, guiding personalized care, and encouraging engagement.”* |
| 2. The **conclusion** is informative but slightly overstated. It should reflect that HRQOL measurement did not improve HRQOL or pain, but was linked to improved enablement | We have changed the conclusion to:  “*Routine measurement of HRQOL by the e-EQ-5D-5L did not improve HRQOL or pain, but was associated with better self-care enablement in primary care patients with chronic knee/back problem*s.” |
| **Introduction**  3. The introduction provides a clear and logical background, but it could be strengthened by explicitly highlighting the knowledge gap i.e., although HRQOL measures are known to enhance patient–doctor communication, their actual clinical impact on outcomes in primary care remains uncertain | We have made this point more explicit.  *“However, despite the potential benefits, routine measurement and reporting of HRQOL is rarely implemented in real clinical practice due to uncertainty on the clinical benefit and logistic barriers of time and workload, leaving its clinical impact unclear.”* (Paragraph 2, p.4) |
| 4. The definition of patient enablement is appropriate; still, linking it more explicitly to HRQOL improvement and chronic disease self-management would make the argument more cohesive. | We have revised the paragraph to be stronger with this point explicitly pointed out.  “*It is particularly important for progressive conditions such as chronic knee/back problems, where patient education and self-care are essential. By fostering greater understanding and coping ability, patient enablement may moderate the impact of pain on HRQOL (24) and serve as a critical link between HRQOL improvement and chronic disease self-management*.” (last paragraph p..4) |
| 5. Minor language refinements e.g., “to facilitate routine HRQOL measurement in busy primary care clinics, our team developed…” instead of “to facilitate routine HRQOL measurement in busy primary care clinics our research team has developed” would improve readability. | Thank you for the suggestion. We have made this change (p.4) and checked the grammar of the whole manuscript to improve readability. |
| **Method**  6. The study design is appropriate (cluster RCT, 12 months, single-blind), but it would be useful to explain *why* the cluster design was chosen and whether intra-cluster correlation was accounted for in the sample size calculation and analysis. | We chose a cluster, instead of individual, randomization design to reduce the chance of contamination among participants recruited from the same clinic.  Yes, we accounted for intra-cluster correlation (ICC) in the sample size calculation. We have added the relevant information to the Sample size calculation section (p.6-7) of the revised manuscript.  We did not adjust for ICC in the main analysis according to the original protocol. To address the reviewer’s comment, we carried out a sensitivity analysis with a GEE with correction of clustering effect, which showed similar correlations between the intervention and the WOMAC and the PEI-2 scores, although the effect on PEI-2 score did not reach statistical significance.  We have added the relevant information to the Results section (paragraph 1 and 5, p.18) of the revised manuscript, and revised supplementary files (S6 Table S3). We have also pointed out the limitation of the results in the Discussion section (paragraph 1, p.24) in the revised manuscript. |
| 7. Please clarify what “single-blind” refers to participants, doctors, or outcome assessors | Single-blind refers to the outcome assessors who administered the questionnaire by telephone during follow-up. (Paragraph 1, p.6) |
| 8. The target sample size (n=1374) is reported, but the **sample size calculation formula and assumptions** (effect size, power, ICC, etc.) are not shown here. | We have described the details of the sample size calculation and added the formulae in the revised manuscript (Paragraph 2 p.6 and paragraph 1, p.7). |
| 9. Randomisation at the clinic level is appropriate, but additional detail on allocation concealment and steps taken to minimize selection bias before recruitment would be helpful | We have provided additional information on allocation concealment in the revised manuscript. (Paragraph 1, p.6).  *“For allocation concealment, we concealed the names of the clinics and allocated them with a number, ranging from 01 to 06, randomly, prior to randomisation to minimise bias.”* |
| 10. Since only six clinics were included, please discuss whether stratification or matching was performed to balance clinic characteristics between groups. | In this RCT, we did not include any stratification/matching to balance clinic characteristics, and we have pointed out this possible limitation in the revised manuscript (paragraph 1, p.24).  *“First, only six clinics were included, and we did not perform any stratification or matching to balance clinic characteristics between groups, which might have led to bias despite randomization.”* |
| 11. Please clarify whether doctors in the intervention group received training or guidance on how to interpret and use HRQOL reports during consultations. | Yes, all doctors in the intervention clinics were given a training session on how to interpret the report on EQ-5D-5L HRQOL profile and VAS scores. We have added this information to the revised manuscript (last paragraph, p.7).  “*All doctors in the intervention clinics received a training session on the interpretation of the EQ-5D-5L HRQOL profile and VAS scores.”* |
| 12. The schedule for baseline and follow-up assessments is well defined but consider clarifying whether the same interviewer conducted all follow-ups and whether inter-rater reliability was assessed. | The baseline interview and follow-up interviews were carried out by different interviewers with the latter blinded to the group allocation. All interviewers received training in standardised administration of the questionnaires, but we did not specifically test for inter-rater reliability. We have explained this more clearly in the revised manuscript (paragraph 3, p.8)  *“To ensure consistency for data collection, all interviewers, both face-to-face and telephone interviewers, had been trained by the study investigators and were given a standardised script to follow verbally during interviews.”* |
| 13. The data collection method (telephone vs face-to-face) differs between baseline and follow-ups; discuss any potential mode effect on data consistency. | We agree that there could be potential mode effect on data consistency, but we believe the bias would be small since the interviewers were trained and previous studies have confirmed inter-rater reliability between face-to-face and telephone interview modes. We have pointed out this limitation (paragraph 1, p. 24) |
| 14. The use of multiple PROMs is appropriate, but the manuscript should explain whether any correction for multiple comparisons was applied to avoid Type I error inflation. | We noted the reviewer’s comment on the possibility of a type I error. We did not apply any correction for multiple comparisons according to the original protocol that was published, but we believe the likelihood of a type I error is small since the most p values were <0.01. |
| 15. Clarify how missing data were handled | We have clarified in the revised manuscript (last paragraph p.9).  “*Multiple imputations were used to substitute missing data.*” |
| 16. The authors should state whether analyses were **intention-to-treat (ITT)** or **per-protocol**. | We have clarified in the revised manuscript (last paragraph p.9).  *“All data were analysed by intention to treat, in which participants who were randomised and had completed at least one repeat outcome measurement was included in the analysis.”* |
| **Results**  17. The loss to follow-up rate is acceptable, but it should be noted whether attrition was statistically compared between groups and if missing data could have introduced bias. | We have supplemented a comparison of the characteristics between the participants’ who had and had not completed follow-up in the Results (paragraph 2, p.10) of the revised manuscript and revised supplementary file (supplementary Table S1). |
| 18. The text mentions *no significant difference in PROMs at baseline*, which supports group comparability; however, please specify whether these comparisons were performed using appropriate tests adjusted for clustering. | Baseline comparisons were conducted using the chi-square test. No adjustment for clustering was applied to the analysis, in adherence to the original protocol that was published. |
| 19. Clarify whether participants with both knee and back problems were analyzed as a combined group or separately. | Participants with both knee and back problems were analysed as a combined group (last paragraph, p.9). |
| 20. Although the tables are referenced, the narrative could briefly report key numerical values (e.g., mean changes and confidence intervals) rather than relying mainly on p-values. | Mean changes and 95% confidence interval are now supplemented in the text in the revised manuscript. |
| 21. Effect sizes and clinical significance should be discussed, particularly for WOMAC and PEI-2 outcomes. | The effect sizes of within group changes (IG:0.16; CG: 0.02) and that of between group difference in changes (0.13) of the WOMAC total score after 12 months were small, which might not be clinically important. On the other hand, the effect size of between group difference in changes in PEI-2 score after 12 months was moderate (0.30), which falls within the range of 0.2 to 0.5 that is associated with a minimal clinically important difference (MCID) for PROM reported in the literature. The information on effect size is added to the Data analysis (paragraph 1, p.10), Results (paragraphs 2 and 3, p.17) and Discussion (paragraphs 2 and 3, p. 21) of the revised manuscript. |
| 22. The authors mention using GEE models, but it would be useful to specify whether they accounted for clustering by clinic and adjusted for baseline covariates. | We did not adjust for ICC in the main analysis according to the original protocol. To address the reviewer’s comment, we carried out a sensitivity analysis with a GEE with correction of clustering effect, which showed similar correlations between the intervention and the WOMAC and the PEI-2 scores, although the effect on PEI-2 score did not reach statistical significance.  We have added the relevant information to the Results section (paragraph 3 and 5, p.17) of the revised manuscript, and revised supplementary files (S6 Table S3). We have also pointed out the limitation of the results in the Discussion section (paragraph 3, p.21) in the revised manuscript. |
| 23. The section could be streamlined by avoiding repetitive mentions of significance for each follow-up. | Thank you for the suggestion, the section has been streamlined for more clarity. |
| **Discussion**  24. The discussion could be strengthened by more explicitly addressing the clinical relevance of the observed improvements in enablement scores for instance, whether the magnitude of change in PEI-2 is meaningful in real-world practice. | Thank you for your insightful comment regarding the clinical relevance of changes in PEI-2 enablement scores. There is currently no formally validated minimal clinically important difference (MCID) for PEI-2, but an effect size of 0.3, as explained in our response to comment 21, lies within the range of 0.2 to 0.5 associated with a MCID for PROM reported in the literature. We have elaborated on the clinical significance of the PEI-2 score in the revised manuscript (paragraph 3, p.21). |
| 25. While the authors suggest that HRQOL reporting may improve doctor awareness of psychosocial issues, this point could be expanded with reference to behavioral or patient engagement theories e.g., patient activation, shared decision-making. This would enhance the conceptual grounding of the discussion. | We thank the reviewer for the suggestion.  We have included definitions and pathways to patient-centred care through the concepts of enablement, shared decision making and patient activation in the Discussion of the revised manuscript (paragraph 2, p.22). |
| 26. The limitations section is concise but could be expanded. Specifically, the impact of COVID-19 restrictions on both recruitment and outcomes deserves more emphasis, as it may have introduced systematic bias or affected patient behaviors differently across clusters. | We have added:  *“Fourth, the study was conducted during COVID-19 restrictions, which may have influenced patients’ physical activity, access to healthcare, and health behaviours, potentially confounding outcomes*.” (paragraph 1, p.24) |
| 27. The comparison with prior studies could benefit from a deeper discussion on why previous interventions showed improvements in mental health outcomes and why this trial did not translate that effect into HRQOL or pain reduction. | We have reorganized this section to be more deliberate in our explanation of why this trial did not translate effects into HRQOL or pain reductions in the revised manuscript (p24-25). |
| 28. Future research directions could be strengthened by suggesting mixed-methods evaluations or cost-effectiveness analyses to determine the broader value of routine HRQOL measurement. | We thank the reviewer for the suggestion.  We have added more discussion on future mixed methods and cost-effectiveness studies in the revised manuscript (paragraph 2, p.24. |
| **Conclusion**  29. A closing remark highlighting the potential policy or primary care implications e.g., supporting personalized management plans would enhance the practical significance of the work. | We have added a Conclusion highlighting the potential policy implications while also being cautious of overstating those implications. (p.26) |

Reviewer 2:

| Reviewer Comment | Response |
| --- | --- |
| This manuscript describes a cluster randomised controlled trial investigating the impact of routine electronic HRQOL measurement (using the e-EQ-5D-5L) on patient-reported outcomes in primary care patients with chronic knee/back problems. The study found that the intervention did not improve the primary outcome of disease-specific HRQOL (WOMAC) or pain but was associated with a statistically significant improvement in patient self-care enablement (PEI-2). The overall reporting quality is good, but several key items require clarification. Authors should address these. | We thank the reviewer for the endorsement. |
| 1. The manuscript should explicitly state whether there were any changes to the trial methods or primary/secondary outcomes after commencement, relative to the published protocol. No information is provided on whether there were any changes to methods after trial commencement (e.g., to eligibility criteria, outcomes) from the published protocol. | Thank you for raising this issue. We have clarified such in our revised method section.  *“No changes to methods were applied after trial commencement.”* (p.5) |
| 2. The planned sample size is mentioned, but the justification (power, alpha, effect size) is not provided in the manuscript. The parameters used for the sample size calculation (alpha, power, effect size, ICC) should be provided. | We have added the details on sample size calculation in the revised manuscript (p.6-7). |
| 3. The manuscript states randomisation was performed at clinic level by a statistician not involved in recruitment, but sequence generation method, allocation concealment, and whether stratification or restricted randomisation were used are not specified. | We have added more details on allocation concealment in the revised manuscript (p.6).  *“For allocation concealment, we concealed the names of the clinics and allocated them with a number, ranging from 01 to 06, randomly, prior to randomisation to minimise bias.”*  In this RCT, we did not include any stratification/matching of in the group allocation, and we have pointed out this limitation in the revised manuscript (paragraph 1, p.24). |
| 4. Only six clusters (3 intervention / 3 control) were randomised. With so few clusters, standard large-sample GEE variance estimates may be unreliable and type I error rates can be inflated. The manuscript does not discuss how the small number of clusters was handled in sample size calculation, analysis, or degrees-of-freedom adjustments. | To adjust for the clustering effect from six clinics, we applied an intra-cluster-coefficient in the sample size calculation to include a larger sample size of 1098 (549 patients per group, and 183 subjects per clinic).  We have supplemented the sample size calculation (paragraph 1, p.7) and pointed out the limitation of the small number of clinics (paragraph 1, p.24) in the revised manuscript.  . |
| 5. Many baseline variables are presented at individual level (Table 1). Because randomisation occurred at cluster level, cluster-level baseline summaries and tests (or at least ICCs and cluster means) should be reported. The manuscript notes some baseline differences in healthcare use (e.g., oral prescribed medications, self-medication) which could confound post-randomisation management decisions. | We have supplemented a statistical comparison on participants’ characteristics between different clusters, with ICC and cluster means, in the revised supplementary file (supplementary table S2, p.10-14). |
| 6. Settings are described, but more detail on the type of primary care clinics (e.g., patient demographics, urban/rural) would be helpful | We have clarified the types of primary care clinics and the respective patient demographics that were included in our study in the revised manuscript (1st paragraph, p 6).  *“Six urban public primary care clinics, from two out of the seven geographic clusters organised by the Hospital Authority in Hong Kong, were randomised into the intervention group (IG) and control group (CG) by simple randomisation, with three clinics in each group. Service users in public primary care clinics in Hong Kong mainly included the older adultsm, low-income individuals, nd patients with chronic diseases. ”* |
| 7. A statement on the monitoring and reporting of any adverse events is required, even if none occurred. | We have included a statement on monitoring and reporting of adverse events in our revised manuscript (paragraph 2, p.8).  “*This study was considered to be a low-risk trial where both the intervention and control groups received their usual medical care. Rules for early stopping were not applicable as the clinicians involved in this study were not blinded to intervention allocation. Collection and assessment of reported adverse events and other unintended effects of the trial interventions or trial conduct were performed on a continuous basis, though none occurred throughout the trial. All unintended effects and adverse events were planned to be reported every 6months to the Institutional Review Board (IRB) of the University of Hong Kong and Queen Mary Hospital*.” |
| 8. Attrition is reported (10.6% IG, 8.0% CG). There is no clear description of how missing data were handled (complete-case vs. imputation), whether missingness was balanced, or whether intention-to-treat (ITT) principle at the individual level was followed. | Multiple imputations were used to substitute missing data. All data were analysed by intention to treat, in which participants who were randomised and had completed at least one repeat outcome measurement was included in the analysis. (last paragraph, p.9) |
| 9. For the positive finding on patient enablement (PEI-2), a discussion of the Minimal Clinically Important Difference (MCID) would help interpret the practical significance of the result. | There is currently no formally validated minimal clinically important difference (MCID) for PEI-2, so we can only take reference to the MCID associated with PROM reported in the literature. The effect size of the between group difference in changes in PEI-2 score after 12 months was medium (0.30), which falls within MCID range for PROM. The information is added to the Results (p.17) Discussion section (p. 21). of the revised manuscript. |
| 10. Primary outcome was WOMAC (range 0–96). The observed adjusted difference at 12 months was β=2.43 (worse in IG) and p=0.018. It is not clear whether this difference is clinically meaningful; authors do not discuss minimal clinically important difference (MCID) for WOMAC or place effect sizes in clinical context. | The effect sizes of within group changes (IG:0.16; CG: 0.02) and that of between group difference in changes (0.13) of the WOMAC total score after 12 months were smaller than the range of 0.2 -0.5 range of MCID for PROMS reported in the literature. We have added the information on effect sizes in the Results (p.17) and discussed the clinical significance (p.21) in the revised manuscript. |
| 11. The Discussion is clear but limited in scope. It would benefit from a more comprehensive analysis of the findings within a broader clinical and digital-health context. At present, it mainly restates results without sufficiently exploring mechanisms, clinical relevance, or implications for practice. I recommend the authors substantially expand this section to: | We have reorganized this section to be more deliberate in our explanation on why this trial did not translate effects into HRQOL or pain reductions, the mechanism on how HRQOL data can trigger patient enablement, shared decision making and patient activation as a pathway to patient-centred care and implications for future research and practice in the revised manuscript (p.21-22). |
| - Explain why EQ-5D-5L feedback may have improved patient enablement but not pain or function, considering possible behavioural and communication mechanisms. | We have added additional explanations including behavioural and communication methods in the discussion. (p.23) |
| - Interpret the magnitude of change in WOMAC relative to the established minimal clinically important difference (MCID) to clarify whether statistically significant differences are also clinically meaningful. | We have added the following discussion to the revised manuscript (p.21)  *“This study found that HRQOL measured by WOMAC worsened over 12 months in both intervention and control groups, and the change was significantly greater in the IG than CG. However, the effect sizes of within group changes (IG:0.16; CG: 0.02) and that of between group difference in changes (0.13) of the WOMAC total score were smaller than the range of 0.2 -0.5 range of the minimal clinically important difference (MCID) for patient reported outcome measures (PROM) reported in the literature. Therefore the difference might not be clinically important”* |
| -Acknowledge and discuss key methodological constraints, including the very small number of clusters, potential baseline imbalances, possible contamination, the limited generalisability beyond Hong Kong public primary care, and the appropriateness of using WOMAC for participants with back pain. | We have added these points to the limitations section (paragraph 1, p.24). |
| - Compare the results with previous trials or systematic reviews on digital or electronic PROM feedback interventions in primary care | We have included a more detailed review of the literature on the feasibility, acceptability and effectiveness of electronic PROM in primary care in the revised manuscript (p.20 to p.21). |
| - Discuss how PROM feedback could be integrated into clinical workflows, electronic health records, etc | We have elaborated on how PROM feedback could be integrated into clinical practice in the revised manuscript (p25). |
| - The current conclusion somewhat overstates the benefits of the intervention. It should be more balanced. | We have made the conclusion more balanced in the revised manuscript (last paragraph, p.25). |

Reviewer # 3

| Reviewer Comment | Response |
| --- | --- |
| The article presents the effectiveness of routine measurement of HRQOL in improving patient-reported outcomes in primary care patients with chronic knee and back problems. The authors draw on existing literature and research from the field of HRQOL research and present the results clearly and concisely. | We thank the reviewer for the endorsement. |
| The abstract and Background section is well written. The Background section provides relevant and necessary information to the study and an insight in relevant previous research. The identified research gap and what this study adds to the existing body of knowledge is described and discussed. The context is described and is helpful in understanding the study purpose. | We thank the reviewer for the endorsement |
| The authors provide new insights into how routine measurement of HRQOL may improve self-care and widen the range of treatments of chronic knee and back problems. The analysis and comparisions beween the IG ang CG are sufficiently described, detailed and clearly presented. The longitudinal design provide additional insights into development of the conditions and HRQOL. | We thank the reviewer for the endorsement |
